# Forensic efficiency and genetic variation of 30 InDels in Vietnamese and Nigerian populations

**DOI:** 10.18632/oncotarget.21494

**Published:** 2017-10-04

**Authors:** Weian Du, Zhiyong Peng, Chunlei Feng, Bofeng Zhu, Bangchao Wang, Yue Wang, Chao Liu, Ling Chen

**Affiliations:** ^1^ School of Forensic Medicine, Southern Medical University, Guangzhou 510515, China; ^2^ Nanfang Hospital, Southern Medical University/The First School of Clinical Medicine, Southern Medical University, Guangzhou 510515, China; ^3^ Guangzhou Forensic Science Institute, Guangdong Province Key Laboratory of Forensic Genetics, Guangzhou 510030, China; ^4^ Guangdong Homy Genetics Incorporation, Foshan 512000, China

**Keywords:** insertion/deletion loci, DIPplex, Vietnamese, Nigerian, forensic genetics

## Abstract

Insertion/deletion polymorphisms (InDels) are ubiquitous diallelic genetic markers that have drawn increasing attention from forensic researchers. Here, we investigated 30 InDel loci in Vietnamese and Nigerian populations and evaluated their usefulness in forensic genetics. The polymorphic information content of these populations ranged, respectively, from 0.164 to 0.375 and from 0.090 to 0.375 across loci. After Bonferroni correction, no significant deviation from Hardy-Weinberg equilibrium was found, except for HLD97 in the Nigerian population. The cumulative power of exclusion for all 30 loci in the Vietnamese and Nigerian populations was 0.9870 and 0.9676, respectively, indicating that this InDel set is not suitable for paternity testing in these populations, but could be included as a supplement. For the Vietnamese and the Nigerian populations, the mean observed heterozygosity was 0.5917 and 0.6268, and the combined discrimination power of the 30 loci was 0.9999999999767 and 0.9999999999603, respectively. These findings indicated that these InDels may be suitable for personal forensic identification in the studied populations. The results of *D*_A_ distance, phylogenetic tree, principal component, and cluster analyses were consistent and indicated a clear pattern of regional distribution. Moreover, the Vietnamese population was shown to have close genetic relationships with the Guangdong Han and Shanghai Han populations.

## INTRODUCTION

Typing of short tandem repeat (STR) loci is the most effective and widely used identification method in the forensic field. The advantages of analyzing STRs are that STRs are multi-allelic, highly heterozygous, and easy to analyze [1]. However, limitations of the method are STRs’ high mutation rates and long amplicon sizes unsuitable for degraded DNA [2, 3]. In recent years, insertion/deletion polymorphisms (InDels) emerged as promising genetic markers that have attracted considerable attention in the field of forensic research. InDels are short-length diallelic polymorphisms derived from single mutations that are widely represented across the human genome. Compared with other genetic markers, InDels have prominent advantages such as lower mutation rates, small amplicon sizes, no stutter peaks, and simplicity to implement in forensic genetic laboratories [4]. Furthermore, they are suitable for biogeographical research, testing of highly degraded DNA, and can help identify the source of mutations in STR loci [5, 6].

Guangdong Province is a large economic and trade hub in China, with more than one million foreigners. Nigerians constitute the major expatriate community in Guangdong. Vietnam is adjacent to Guangxi, which enables easy migration into Guangdong for work or marriage. Consequently, a number of cases relating to these highlighted populations require forensic DNA analysis in Guangdong.

The Qiagen Investigator DIPplex kit (Qiagen, Hilden, Germany) is the first commercially available kit that allows simultaneous amplification and identification of 70–160 base pair amplicons of 30 InDels. Information on localization and motif for the 30 InDels is shown in Supplementary Table 1. This kit has been used to investigate, among others, several Asian, European, and American populations, and has been alternatively confirmed as a potential extension for STR kits, a separate and effective system for individual identification, and a supplementary kit for paternity testing [7–17]. However, to date, no investigations using this method have been performed in Vietnamese and Nigerian populations. In this study, the Qiagen Investigator DIPplex kit was used to acquire Vietnamese and Nigerian population data, evaluate the forensic value of 30 InDels, and analyze genetic differences between the studied populations and other reported populations.

## RESULTS AND DISCUSSION

**Figure 1:**
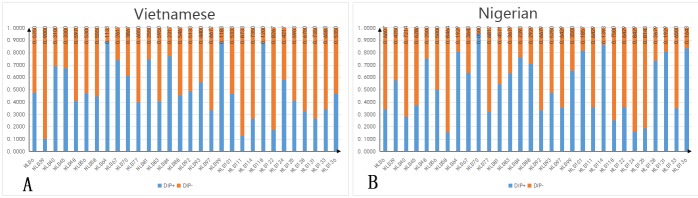
Allele frequency distribution Data from 30 InDel loci in Vietnamese (n = 300) and Nigerian (n = 140) populations. HLD, human locus deletion/insertion polymorphism; DIP+, long allele frequency; DIP-, short allele frequency. **(A)** Allele frequency distribution in Vietnamese; **(B)** allele frequency distribution in Nigerian.

**Figure 2:**
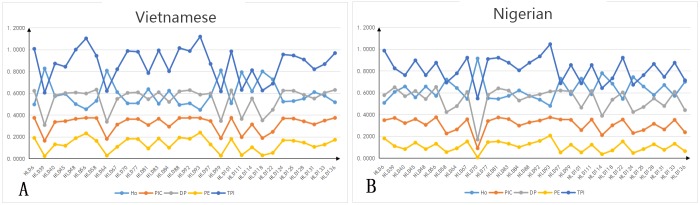
Forensic statistical parameters Data from 30 InDel loci in Vietnamese (n = 300) and Nigerian (n = 140) populations. HLD, human locus deletion/insertion polymorphism; Ho, observed heterozygosity; PIC, polymorphic information content; PE, power of exclusion; DP, discrimination power; TPI, typical paternity index. **(A)** Forensic statistical parameters in Vietnamese; **(B)** forensic statistical parameters in Nigerian.

The allele frequencies and forensic statistical parameters of the 30 InDel loci analyzed in the Vietnamese and Nigerian populations are shown in Supplementary Table 2 and Figures 1 and 2. The frequencies of the short allele ranged from 0.1115 (HLD64 locus) to 0.9000 (HLD39 locus) in the Vietnamese population, and from 0.0500 (HLD70 locus) to 0.8464 (HLD58 locus) in the Nigerian population. The observed heterozygosities (Ho) for both Vietnamese (range 0.447–0.827) and Nigerian (range 0.479–0.914) samples were over 0.3. The polymorphic information content (PIC) of the Vietnamese samples ranged from 0.164 (HLD39) to 0.375 (HLD92), with 77% (23 out of 30) being over 0.3. The PIC of the Nigerian samples ranged from 0.090 (HLD70) to 0.375 (HLD56), with 67% (20 out of 30) being over 0.3. This shows that most of the InDels are highly polymorphic. After Bonferroni correction (*p* > 0.05/30 = 0.0017), no significant deviation from Hardy-Weinberg equilibrium was found, except for HLD97 (*p* = 0.00001) in the Nigerian population, which may be due to the limited sampling size. The cumulative power of exclusion (PE) of the 30 InDels was 0.9870 in the Vietnamese and 0.9676 in the Nigerian population. Because both values were less than 0.9999, this InDels set was not powerful enough to perform paternity testing in these populations. However, the combined discrimination power (DP) of these loci the Vietnamese and Nigerian populations was 0.9999999999767 and 0.9999999999603, respectively, indicating that the InDels set was effective for individual identification in these populations.

**Figure 3:**
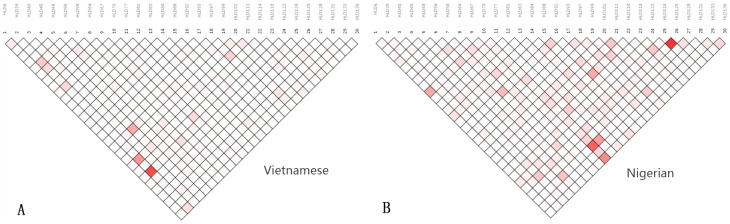
Linkage disequilibrium patterns in the Vietnamese and Nigerian populations **(A)** Linkage disequilibrium patterns in Vietnamese; **(B)** linkage disequilibrium patterns in Nigerian.

Linkage disequilibrium (LD) was analyzed by SNP Analyzer v2.0 for each population separately, and the results are shown in Figure 3. The intensity of the red color in the plot determines the strength of linkage between loci. Positive linkage, represented by a thick black line, was absent among the 30 InDel loci within the two study populations. Moreover, the level of LD between the 30 InDel loci was estimated using r^2^ and tested by the SNPAnalyzer program. The r^2^ values of all loci were less than 0.2, indicating that subsequent analyses should treat the 30 InDels as independent loci in these two populations.

**Figure 4:**
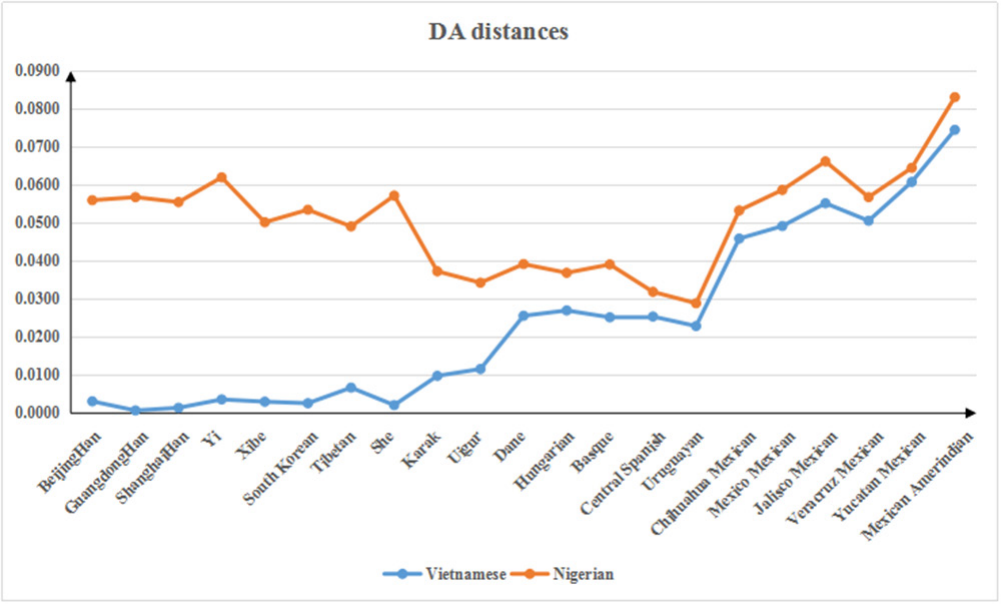
Genetic distances among the Vietnamese and Nigerian populations and 21 reference populations

Genetic distances *(D*_*A*_ distance) were calculated by the DISPAN program and used to compare the Vietnamese and Nigerian populations with 21 other reference populations including Beijing Han [7], Guangdong Han [8], Shanghai Han [9], Yi [10], Xibe [11], South Korean [12], Tibetan [7], Kazak [7], Uighur [7], Dane [13], Hungarian [14], Basque [14], Central Spanish [14], Uruguayan [15], She [16], and six populations of Mexican origin [17] (Supplementary Table 3 and Figure 4). The Vietnamese population was found to have a close genetic distance with most Asian populations, especially the Guangdong Han and Shanghai Han populations. Meanwhile, a relatively large genetic distance was observed between the Nigerian population and the other populations. For both Vietnamese and Nigerian populations, the line chart suggested high consistency between the genetic and geographical distances.

**Figure 5:**
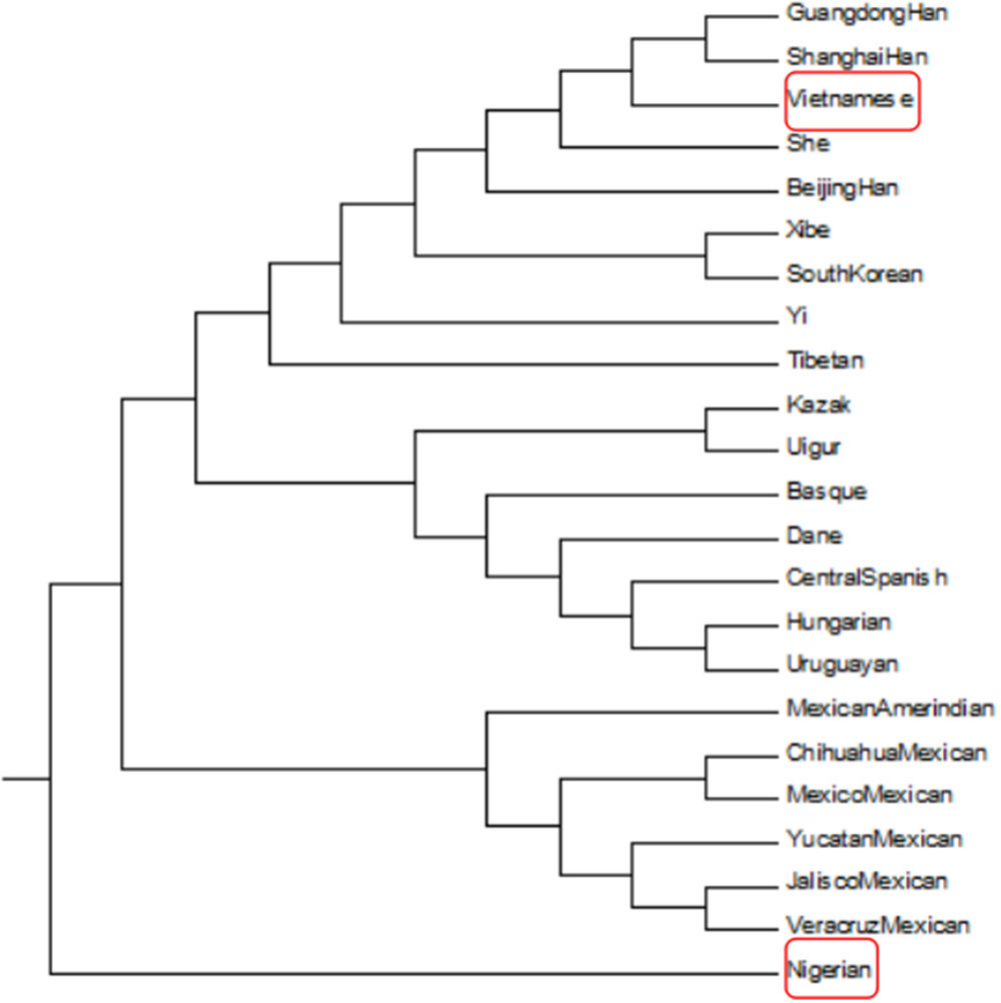
Phylogenetic tree constructed based on DA distances

A phylogenetic tree generated based on *D*_*A*_ distance showed population clusters corresponding to continental regions (Figure 5). The Nigerian population was the only one of African origin among the 23 populations represented, and was clustered on one branch. The other primary branch divided into two main branches: one included 6 North American populations (Chihuahua Mexican, Mexico Mexican, Jalisco Mexican, Veracruz Mexican, Mexican Amerindian, and Yucatan Mexican), and the other further diverged into two branches: one containing 9 East Asian populations (Vietnamese, Guangdong Han, Shanghai Han, She, Beijing Han, Xibe, South Korean, Yi, and Tibetan) and the other containing 2 Eurasian populations (Kazak and Uighur), 4 European populations (Basque, Dane, Central Spanish, Hungarian) and a South American population (Uruguayan). These results revealed that the Asian population was genetically closer to the Eurasian and European populations than to the North American populations, and most distant from the African population. As in the *D*_*A*_ distance plot, the phylogenetic analysis also showed that the Vietnamese population was closely related to the Guangdong Han and Shanghai Han populations. A recent study has shown that the Vietnamese population in Yunnan has a close relationship with the Yunnan Miao and Guizhou Miao populations [18]. Vietnam is located in the eastern part of the Central South Peninsula of Southeast Asia and shares borders with Guangxi and Yunnan in the southwest of China. Vietnam was under the rule of China from 111 BC until 938 AD. Chen Zhongjin, a famous Vietnamese historian, reported that Vietnam imported a large amount of Chinese culture and was deeply influenced by China [19]. Thus, both geographical and historical factors may help explain why the Vietnamese population has close genetic relations with the Chinese.

**Figure 6:**
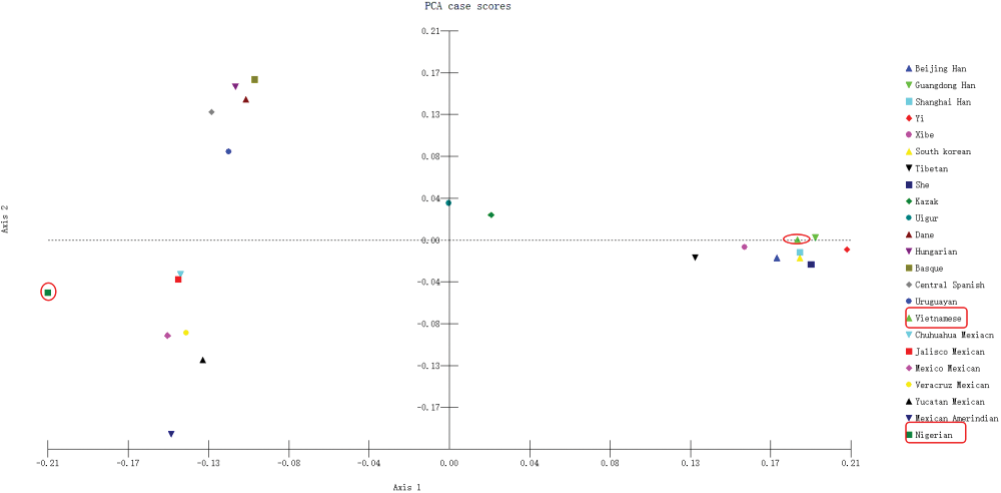
PCA based on 30 InDel loci in the Vietnamese and Nigerian populations and 21 reference populations

Principal component analysis (PCA) was conducted by MATLAB 2007a based on allele frequencies of our Vietnamese and Nigerian populations, as well as 21 other reference population groups. As shown in Figure 6, the results revealed a clear regional distribution pattern. Nine East Asian groups, including the Vietnamese, were located on the right of the chart, while the Kazak and the Uighur were located centrally. Four European groups were located on the upper left quadrant and close to the Uruguayan group. The lower left quadrant included the 6 North American groups as well as the Nigerian group, which was located further to the left. Here again, the Vietnamese population’s closest relationships were with the Guangdong Han and Shanghai Han populations. Thus, PCA results were in accordance with phylogenetic tree results.

**Figure 7:**
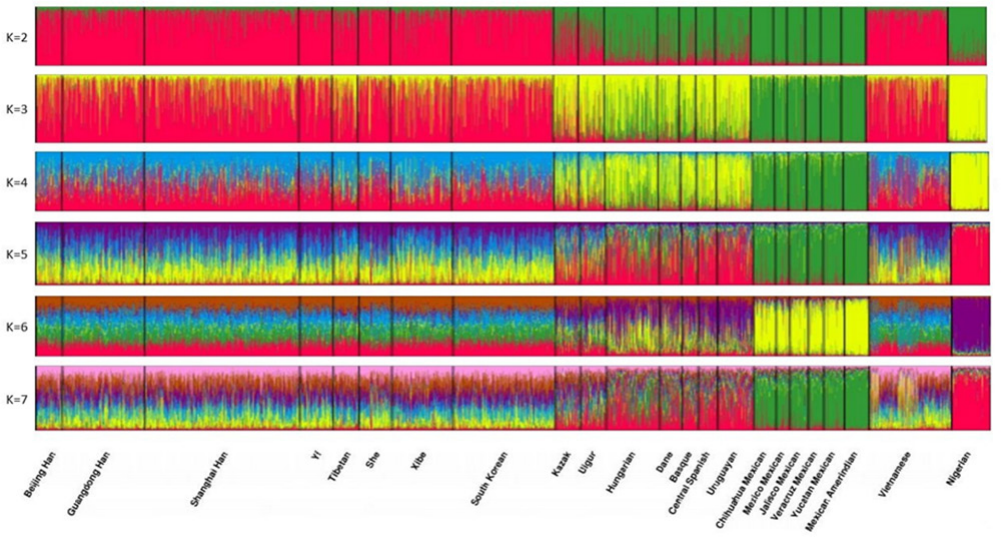
Clustering analysis by STRUCTURE The full-loci dataset was analyzed, assuming K = 2-7.

Cluster analysis was performed using the STRUCTURE program to assess the ancestry predictive value of the 30 InDels (Figure 7). From this analysis, 23 populations were classified into geographic patterns. At K = 2, the Vietnamese and the other 8 East Asian populations were predominantly composed of red component, while the 6 North American populations were predominantly composed of green component. Furthermore, both red and green components were observed in the 2 Eurasian populations, which displayed a higher proportion of red component, and in the African population and the 5 European populations, which displayed a higher proportion of green component. At K > 2, the Vietnamese population was comparable with the other 8 East Asian populations, evidencing the aforementioned close relationships between these populations. At K = 3–4, the African population was unique in component color. At K = 3–4, the 5 European populations were composed of a higher proportion of yellow component, similar to the African population. This result suggested that the European population likely originated in Africa; however, this result warrants further investigation.

## MATERIALS AND METHODS

### Sample collections and DNA extraction

Bloodstain samples were collected from 300 unrelated Vietnamese and 140 unrelated Nigerian individuals. All study participants had recently resided in China, were older than 20 years of age, and both their parents and grandparents were of specific aboriginal descent. Before inclusion in the study, all participants gave full informed consent. The Chelex-100 method was employed to extract template DNA from the samples, following the procedure described by Walsh et al. [20].

### InDel genotyping

The extracted DNA was amplified by the Investigator DIPplex kit in the GeneAmp 9700 PCR System (Applied Biosystems Inc., CA, USA) following manufacturer’s instructions. PCR products were detected by capillary electrophoresis in an ABI 3130xl Genetic Analyzer (Applied Biosystems Inc.) using an SST-BTO size standard and reference allelic ladder provided in the Investigator DIPplex kit. Data analysis and genotyping were performed by GeneMapper ID v3.2 software (Applied Biosystems Inc.). Internal controls (negative control and 9948 male DNA positive control) were genotyped with each sample batch to ensure accuracy of the results.

### Quality control

The study analyzed DNA polymorphisms in accordance with the recommendations of the International Society for Forensic Genetics (ISFG), as described by Schneider [21].

### Statistical analyses

Allele frequencies, Hardy-Weinberg equilibrium, and forensic statistical parameters such as Ho, PIC, PE, DP, and TPI were calculated using modified PowerStats v1.2 software (Promega, WI, USA). The DISPAN program was used to estimate *D*_*A*_ distances [22]. SNP Analyzer v2.0 was used to analyze LD [23]. Population structure was analyzed with STRUCTURE v2.2. Principal component analysis (PCA) was performed based on allele frequencies in MATLAB 2007a (MathWorks Inc., USA). A phylogenetic tree based on *D*_*A*_ distances with 1000 bootstrap replications was mapped using MEGA v5.

## CONCLUSIONS

We report here, for the first time, the forensic efficiency and genetic variation of 30 InDels in Vietnamese and Nigerian populations. Although these InDels showed great potential for forensic personal identification in those populations, we conclude that they could only be used as a useful supplement to routine STR detection for paternity testing. Analyses of *D*_*A*_ distance, phylogenetic tree, and population principal component were consistent and showed a clear regional distribution of the studied populations. Furthermore, close genetic relationships were identified between the Vietnamese and Guangdong Han and Shanghai Han populations, whereas distant relationships were identified between the Nigerian the other populations analyzed.

## SUPPLEMENTARY MATERIALS TABLES








