# Supplementary material for: Forensic efficiency and genetic variation of 30 InDels in Vietnamese and Nigerian populations
Source: Oncotarget. 2017 Oct 4;8(51):88934–40. doi: 10.18632/oncotarget.21494 (PMC5687658; doi:10.18632/oncotarget.21494)
Supplement: Supplementary file 1 [file oncotarget-08-88934-s001.pdf]

Forensic efficiency and genetic variation of 30 InDels in Vietnamese and Nigerian populations

SUPPLEMENTARY MATERIALS

Supplementary Table 1: Locus-specific information of the Investigator DIPplex Kit

See Supplementary File 1

Supplementary Table 2: Allele frequency distribution and forensic statistical parameters of the 30 INDEL loci in Vietnam group(n=300) and Nigeria group (n=140)

See Supplementary File 2

Supplementary Table 3: The DA distances among the Vietnamese and Nigerian groups and other 21 reference groups

See Supplementary File 3
