# Supplementary material for: Forensic efficiency and genetic variation of 30 InDels in Vietnamese and Nigerian populations
Source: Oncotarget. 2017 Oct 4;8(51):88934–40. doi: 10.18632/oncotarget.21494 (PMC5687658; doi:10.18632/oncotarget.21494)
Supplement: Supplementary file 2 [file oncotarget-08-88934-s002.docx]

Table S1. Locus-specific information of the Investigator DIPplex Kit

| DIP locus | GenBank accession/SNP ID | Chromosome localization | Motif (+DIP) | Reference allele |
| --- | --- | --- | --- | --- |
| HLD77 | rs1611048 | 7q31.1 | TAAG | +DIP |
| HLD45 | rs2307959 | 2q31.1 | CACG | -DIP |
| HLD131 | rs1611001 | 7q36.2 | TGGGCTTATT | +DIP |
| HLD70 | rs2307652 | 6q16.1 | AGCA | -DIP |
| HLD6 | rs1610905 | 16q13 | GCAGGACTGGGCACC | -DIP |
| HLD111 | rs1305047 | 17p11.2 | CACA | -DIP |
| HLD58 | rs1610937 | 5q14.1 | AGGA | +DIP |
| HLD56 | rs2308292 | 4q25 | TAAGT | +DIP |
| HLD118 | rs16438 | 20p11.1 | CCCCA | -DIP |
| HLD92 | rs201771066 | 11q22.2 | GTTT | -DIP |
| HLD93 | rs150042219 | 12q22 | ACTTT | -DIP |
| HLD99 | rs2308163 | 14q23.1 | TGAT | -DIP |
| HLD88 | rs8190570 | 9q22.32 | CCACAAAGA | +DIP |
| HLD101 | rs2307433 | 15q26.1 | GTAG | -DIP |
| HLD67 | rs1305056 | 5q33.2 | CTACTGAC | -DIP |
| HLD83 | rs2308072 | 8p22 | AAGG | -DIP |
| HLD114 | rs2307581 | 17p13.3 | TCCTATTCTACTCTGAAT | -DIP |
| HLD48 | rs28369942 | 2q11.2 | GACTT | -DIP |
| HLD124 | rs6481 | 22q12.3 | GTGGA | -DIP |
| HLD122 | rs8178524 | 21q22.11 | GAAGTCTGAGG | -DIP |
| HLD125 | rs16388 | 22q11.23 | ATTGCC | -DIP |
| HLD64 | rs397832668 | 5q12.3 | GACAAA | +DIP |
| HLD81 | rs17879936 | 7q21.3 | GTAAGCATTGT | -DIP |
| HLD136 | rs16363 | 22q13.1 | TGTTT | -DIP |
| HLD133 | rs2067235 | 3p22.1 | CAACCTGGATT | -DIP |
| HLD97 | rs17238892 | 13q12.3 | AGAGAAAGCTGAAG | -DIP |
| HLD40 | rs146044344 | 1p32.3 | GGGACAGGTGGCCACTAGGAGA | +DIP |
| HLD128 | rs2307924 | 1q31.3 | ATTAAATA | -DIP |
| HLD39 | rs17878444 | 1p22.1 | CCTAAACAAAAATGGGAT | -DIP |
| HLD84 | rs3081400 | 8q24.12 | CTTTC | -DIP |

HLD: Human locus DIP; –DIP: Deletion; +DIP: Insertion.
