# Supplementary material for: Forensic efficiency and genetic variation of 30 InDels in Vietnamese and Nigerian populations
Source: Oncotarget. 2017 Oct 4;8(51):88934–40. doi: 10.18632/oncotarget.21494 (PMC5687658; doi:10.18632/oncotarget.21494)
Supplement: Supplementary file 3 [file oncotarget-08-88934-s003.docx]

Table S2. Allele frequency distribution and forensic statistical parameters of the 30 INDEL loci in Vietnam group(n=300) and Nigeria group (n=140)

| HLD | rs# | Vietnam | | | | | | | Nigeria | | | | | | |
| --- | --- | --- | --- | --- | --- | --- | --- | --- | --- | --- | --- | --- | --- | --- | --- |
|  |  | DIP- | p | Ho | PIC | DP | PE | TPI | DIP- | p | Ho | PIC | DP | PE | TPI |
| 6 | 1610905 | 0.5250 | 0.897 | 0.497 | 0.374 | 0.622 | 0.190 | 1.007 | 0.6607 | 0.307 | 0.507 | 0.348 | 0.577 | 0.181 | 0.986 |
| 39 | 17878444 | 0.9000 | 0.754 | 0.827 | 0.164 | 0.308 | 0.023 | 0.605 | 0.4250 | 0.021 | 0.607 | 0.369 | 0.650 | 0.110 | 0.824 |
| 40 | 2307956 | 0.3100 | 0.948 | 0.573 | 0.336 | 0.581 | 0.131 | 0.872 | 0.7214 | 0.144 | 0.657 | 0.321 | 0.568 | 0.083 | 0.761 |
| 45 | 2307959 | 0.3300 | 0.206 | 0.593 | 0.344 | 0.601 | 0.118 | 0.843 | 0.6286 | 0.541 | 0.557 | 0.358 | 0.616 | 0.142 | 0.897 |
| 48 | 28369942 | 0.5970 | 0.535 | 0.500 | 0.365 | 0.606 | 0.188 | 1.000 | 0.2500 | 0.413 | 0.657 | 0.305 | 0.542 | 0.083 | 0.761 |
| 56 | 2308292 | 0.5300 | 0.099 | 0.453 | 0.374 | 0.597 | 0.232 | 1.103 | 0.5000 | 0.083 | 0.571 | 0.375 | 0.653 | 0.132 | 0.875 |
| 58 | 1610937 | 0.5550 | 0.391 | 0.530 | 0.372 | 0.633 | 0.163 | 0.943 | 0.8464 | 0.634 | 0.721 | 0.226 | 0.422 | 0.055 | 0.693 |
| 64 | 1610935 | 0.1133 | 0.730 | 0.807 | 0.181 | 0.338 | 0.028 | 0.620 | 0.1929 | 0.254 | 0.643 | 0.263 | 0.477 | 0.090 | 0.778 |
| 67 | 1305056 | 0.2617 | 0.916 | 0.610 | 0.312 | 0.548 | 0.108 | 0.820 | 0.3643 | 0.855 | 0.543 | 0.356 | 0.607 | 0.153 | 0.921 |
| 70 | 2307652 | 0.3867 | 0.527 | 0.507 | 0.362 | 0.603 | 0.182 | 0.987 | 0.0500 | 0.698 | 0.914 | 0.090 | 0.170 | 0.006 | 0.547 |
| 77 | 1611048 | 0.6050 | 0.696 | 0.510 | 0.364 | 0.608 | 0.179 | 0.980 | 0.6812 | 0.724 | 0.550 | 0.340 | 0.580 | 0.147 | 0.909 |
| 81 | 17879936 | 0.2550 | 0.538 | 0.637 | 0.308 | 0.545 | 0.093 | 0.785 | 0.4571 | 0.332 | 0.543 | 0.373 | 0.640 | 0.153 | 0.921 |
| 83 | 2308072 | 0.5950 | 0.630 | 0.503 | 0.366 | 0.609 | 0.185 | 0.993 | 0.3679 | 0.342 | 0.571 | 0.358 | 0.620 | 0.132 | 0.875 |
| 84 | 3081400 | 0.2317 | 0.468 | 0.623 | 0.293 | 0.520 | 0.100 | 0.802 | 0.2393 | 0.745 | 0.621 | 0.298 | 0.528 | 0.101 | 0.805 |
| 88 | 8190570 | 0.5467 | 0.724 | 0.493 | 0.373 | 0.617 | 0.193 | 1.014 | 0.2929 | 0.757 | 0.571 | 0.328 | 0.567 | 0.132 | 0.875 |
| 92 | 17174476 | 0.5133 | 0.805 | 0.507 | 0.375 | 0.628 | 0.182 | 0.987 | 0.6679 | 0.650 | 0.536 | 0.345 | 0.585 | 0.158 | 0.933 |
| 93 | 2307570 | 0.4400 | 0.039 | 0.447 | 0.371 | 0.587 | 0.239 | 1.119 | 0.5250 | 0.621 | 0.479 | 0.374 | 0.612 | 0.207 | 1.045 |
| 97 | 17238892 | 0.6672 | 0.485 | 0.575 | 0.345 | 0.598 | 0.130 | 0.869 | 0.6429 | 0.000 | 0.729 | 0.354 | 0.620 | 0.052 | 0.686 |
| 99 | 2308163 | 0.1183 | 0.418 | 0.810 | 0.187 | 0.345 | 0.027 | 0.617 | 0.3500 | 0.314 | 0.586 | 0.351 | 0.612 | 0.123 | 0.854 |
| 101 | 2307433 | 0.5333 | 0.822 | 0.508 | 0.374 | 0.626 | 0.180 | 0.984 | 0.1857 | 0.409 | 0.729 | 0.257 | 0.463 | 0.052 | 0.686 |
| 111 | 1305047 | 0.8733 | 0.533 | 0.793 | 0.197 | 0.364 | 0.032 | 0.630 | 0.6429 | 0.269 | 0.586 | 0.354 | 0.616 | 0.123 | 0.854 |
| 114 | 2307581 | 0.7350 | 0.807 | 0.617 | 0.314 | 0.552 | 0.104 | 0.811 | 0.1393 | 0.595 | 0.779 | 0.211 | 0.388 | 0.036 | 0.642 |
| 118 | 16438 | 0.1200 | 0.624 | 0.800 | 0.189 | 0.351 | 0.030 | 0.625 | 0.7500 | 0.185 | 0.683 | 0.301 | 0.536 | 0.071 | 0.732 |
| 122 | 8178524 | 0.8267 | 0.599 | 0.727 | 0.246 | 0.448 | 0.053 | 0.688 | 0.6429 | 0.930 | 0.543 | 0.354 | 0.603 | 0.153 | 0.921 |
| 124 | 6481 | 0.4217 | 0.681 | 0.523 | 0.369 | 0.624 | 0.168 | 0.955 | 0.8429 | 0.816 | 0.743 | 0.230 | 0.423 | 0.047 | 0.673 |
| 125 | 16388 | 0.5903 | 0.655 | 0.528 | 0.367 | 0.622 | 0.164 | 0.946 | 0.8143 | 0.312 | 0.657 | 0.257 | 0.469 | 0.083 | 0.761 |
| 128 | 2307924 | 0.6750 | 0.714 | 0.550 | 0.342 | 0.585 | 0.147 | 0.909 | 0.2679 | 0.501 | 0.579 | 0.315 | 0.547 | 0.127 | 0.864 |
| 131 | 1611001 | 0.7350 | 0.994 | 0.610 | 0.314 | 0.551 | 0.108 | 0.820 | 0.1929 | 0.681 | 0.671 | 0.263 | 0.478 | 0.076 | 0.745 |
| 133 | 2067235 | 0.6583 | 0.342 | 0.577 | 0.349 | 0.604 | 0.129 | 0.867 | 0.6500 | 0.505 | 0.571 | 0.351 | 0.608 | 0.132 | 0.875 |
| 136 | 16363 | 0.5350 | 0.602 | 0.517 | 0.374 | 0.630 | 0.173 | 0.968 | 0.1643 | 0.518 | 0.700 | 0.237 | 0.440 | 0.064 | 0.714 |

HLD, human locus deletion/insertion polymorphism; DIP-, frequency of short allele; Ho, observed heterozygosity; p, probability value of the exact test for Hardy-Weinberg equilibrium; PIC, polymorphic information content; PE, power of exclusion; DP, discrimination power; TPI, typical paternity index.
