# Supplementary material for: Forensic efficiency and genetic variation of 30 InDels in Vietnamese and Nigerian populations
Source: Oncotarget. 2017 Oct 4;8(51):88934–40. doi: 10.18632/oncotarget.21494 (PMC5687658; doi:10.18632/oncotarget.21494)
Supplement: Supplementary file 4 [file oncotarget-08-88934-s004.docx]

Table S3. The DA distances among the Vietnamese and Nigerian groups and other 21 reference groups

| Groups | BeijingHan | Guangdong Han | Shanghai Han | Yi | Xibe | South Korean | Tibetan | She | Karak | Uigur | Dane | Hungarian | Basque | Central Spanish | Uruguayan | Chihuahua Mexican | Mexico Mexican | Jalisco Mexican | Veracruz Mexican | Yucatan Mexican | Mexican Amerindian | Nigerian |
| --- | --- | --- | --- | --- | --- | --- | --- | --- | --- | --- | --- | --- | --- | --- | --- | --- | --- | --- | --- | --- | --- | --- |
| Guangdong Han | 0.0019 |  |  |  |  |  |  |  |  |  |  |  |  |  |  |  |  |  |  |  |  |  |
| Shanghai Han | 0.0011 | 0.0006 |  |  |  |  |  |  |  |  |  |  |  |  |  |  |  |  |  |  |  |  |
| Yi | 0.0054 | 0.0038 | 0.0040 |  |  |  |  |  |  |  |  |  |  |  |  |  |  |  |  |  |  |  |
| Xibe | 0.0022 | 0.0023 | 0.0015 | 0.0052 |  |  |  |  |  |  |  |  |  |  |  |  |  |  |  |  |  |  |
| South Korean | 0.0024 | 0.0017 | 0.0008 | 0.0042 | 0.0016 |  |  |  |  |  |  |  |  |  |  |  |  |  |  |  |  |  |
| Tibetan | 0.0029 | 0.0055 | 0.0038 | 0.0066 | 0.0037 | 0.0038 |  |  |  |  |  |  |  |  |  |  |  |  |  |  |  |  |
| She | 0.0023 | 0.0015 | 0.0019 | 0.0051 | 0.0032 | 0.0028 | 0.0065 |  |  |  |  |  |  |  |  |  |  |  |  |  |  |  |
| Karak | 0.0083 | 0.0100 | 0.0096 | 0.0133 | 0.0068 | 0.0115 | 0.0074 | 0.0112 |  |  |  |  |  |  |  |  |  |  |  |  |  |  |
| Uigur | 0.0100 | 0.0118 | 0.0114 | 0.0163 | 0.0092 | 0.0135 | 0.0093 | 0.0133 | 0.0013 |  |  |  |  |  |  |  |  |  |  |  |  |  |
| Dane | 0.0251 | 0.0265 | 0.0264 | 0.0315 | 0.0227 | 0.0288 | 0.0226 | 0.0275 | 0.0093 | 0.0083 |  |  |  |  |  |  |  |  |  |  |  |  |
| Hungarian | 0.0255 | 0.0275 | 0.0271 | 0.0325 | 0.0231 | 0.0295 | 0.0222 | 0.0289 | 0.0084 | 0.0068 | 0.0026 |  |  |  |  |  |  |  |  |  |  |  |
| Basque | 0.0270 | 0.0268 | 0.0270 | 0.0328 | 0.0236 | 0.0287 | 0.0258 | 0.0288 | 0.0111 | 0.0096 | 0.0048 | 0.0045 |  |  |  |  |  |  |  |  |  |  |
| Central Spanish | 0.0262 | 0.0269 | 0.0268 | 0.0323 | 0.0226 | 0.0288 | 0.0231 | 0.0285 | 0.0085 | 0.0069 | 0.0030 | 0.0022 | 0.0033 |  |  |  |  |  |  |  |  |  |
| Uruguayan | 0.0230 | 0.0244 | 0.0240 | 0.0286 | 0.0203 | 0.0258 | 0.0199 | 0.0255 | 0.0067 | 0.0057 | 0.0039 | 0.0021 | 0.0043 | 0.0023 |  |  |  |  |  |  |  |  |
| Chihuahua Mexican | 0.0437 | 0.0450 | 0.0448 | 0.0512 | 0.0420 | 0.0484 | 0.0417 | 0.0502 | 0.0260 | 0.0225 | 0.0170 | 0.0137 | 0.0197 | 0.0181 | 0.0189 |  |  |  |  |  |  |  |
| Mexico Mexican | 0.0456 | 0.0483 | 0.0477 | 0.0549 | 0.0462 | 0.0520 | 0.0437 | 0.0543 | 0.0297 | 0.0252 | 0.0200 | 0.0175 | 0.0232 | 0.0220 | 0.0232 | 0.0023 |  |  |  |  |  |  |
| Jalisco Mexican | 0.0534 | 0.0548 | 0.0544 | 0.0602 | 0.0517 | 0.0576 | 0.0502 | 0.0606 | 0.0345 | 0.0313 | 0.0222 | 0.0210 | 0.0256 | 0.0258 | 0.0274 | 0.0041 | 0.0059 |  |  |  |  |  |
| Veracruz Mexican | 0.0498 | 0.0500 | 0.0502 | 0.0566 | 0.0473 | 0.0535 | 0.0468 | 0.0555 | 0.0301 | 0.0263 | 0.0192 | 0.0160 | 0.0209 | 0.0197 | 0.0223 | 0.0024 | 0.0049 | 0.0030 |  |  |  |  |
| Yucatan Mexican | 0.0588 | 0.0599 | 0.0598 | 0.0675 | 0.0569 | 0.0633 | 0.0567 | 0.0655 | 0.0391 | 0.0351 | 0.0267 | 0.0230 | 0.0277 | 0.0279 | 0.0300 | 0.0046 | 0.0066 | 0.0041 | 0.0031 |  |  |  |
| Mexican Amerindian | 0.0718 | 0.0724 | 0.0732 | 0.0798 | 0.0698 | 0.0770 | 0.0698 | 0.0796 | 0.0519 | 0.0477 | 0.0365 | 0.0317 | 0.0389 | 0.0393 | 0.0427 | 0.0090 | 0.0102 | 0.0087 | 0.0064 | 0.0053 |  |  |
| Nigerian | 0.0560 | 0.0568 | 0.0555 | 0.0620 | 0.0502 | 0.0535 | 0.0491 | 0.0572 | 0.0373 | 0.0343 | 0.0392 | 0.0369 | 0.0391 | 0.0319 | 0.0289 | 0.0533 | 0.0587 | 0.0662 | 0.0568 | 0.0645 | 0.0831 |  |
| Vietnamese | 0.0031 | 0.0007 | 0.0014 | 0.0036 | 0.0030 | 0.0026 | 0.0067 | 0.0021 | 0.0098 | 0.0116 | 0.0256 | 0.0270 | 0.0252 | 0.0254 | 0.0229 | 0.0459 | 0.0492 | 0.0552 | 0.0506 | 0.0608 | 0.0745 | 0.0561 |
